# Real-time bowel perfusion monitoring with FUJIFILM ELUXEO® VISION endoscopic imaging system in colorectal surgery

**DOI:** 10.1007/s00464-025-12526-2

**Published:** 2025-12-29

**Authors:** Jordan Martucci, Brian Williams, Aubrey Swinford, Abhinav Gupta, Alexis Peters, Anthony Lim, Sacha Broccard, Kyle G. Cologne, Joongho Shin, Sang W. Lee

**Affiliations:** Division of Colorectal Surgery, Keck Hospital of USC, 1441 Eastlake Avenue, Suite 7418, Los Angeles, CA 90033 USA

**Keywords:** Anastomotic leak, Tissue oxygenation, ELUXEO® VISION, Colon resection, Intraoperative perfusion assessment

## Abstract

**Background:**

Anastomotic leak is a serious complication of bowel anastomosis, with multifactorial etiology. Thorough intraoperative evaluation of the anastomosis using white light endoscopy and leak tests is critical. Indocyanine green fluorescence angiography (ICG-FA) has emerged as a tool to assess bowel perfusion, but has limitations, including patient allergies and procedural complexity. The FUJIFILM ELUXEO® VISION Endoscopic Imaging System uses oxygen saturation imaging (OSI) to measure tissue oxygen saturation (StO_2_) in real time, without requiring dye injection. This study investigates a novel quantitative approach for assessing bowel perfusion endoscopically by evaluating StO_2_ at colon anastomoses.

**Methods:**

The FUJIFILM VISION was used to obtain intraoperative mucosal StO_2_ measurements in 12 patients’ post-colon resection with anastomosis. Measurements were taken at four locations: proximal base, proximal staple line, distal staple line, and distal base. Two-tailed paired *t* tests were used to compare StO_2_ measurements at distant healthy mucosa (base) to the staple line, both proximal and distal to the anastomosis. Patients were followed longitudinally via chart review to monitor outcomes.

**Results:**

The average StO_2_ differences were + 12.58% (95% CI 1.96 – 23.19%) between the proximal base and proximal staple line and + 15.34% (95% CI 6.76 – 23.9%) between the distal base and distal staple line. Significant StO_2_ differences were observed between baseline normal mucosa and staple line adjacent mucosa (proximal: *p* = 0.025; distal: *p* = 0.003). No patients had intraoperative complications or developed anastomotic leaks during the study.

**Conclusions:**

This study shows that the ELUXEO® VISION Endoscopic Imaging System is a potentially useful tool for providing quantitative data on tissue perfusion at and near anastomotic sites, complementing traditional white light endoscopy and leak tests. Larger prospective studies with long-term follow-up are needed to confirm the relationship between StO_2_ measurements and clinical outcomes such as anastomotic leaks and stricture.

**Supplementary Information:**

The online version contains supplementary material available at 10.1007/s00464-025-12526-2.

The tenets of a good bowel anastomosis include tension-free apposition, precise mucosal union, secure water-tight seal, absence of stricture or obstruction, minimal contamination, and adequate blood supply for tissue perfusion [1]. The latter is paramount, especially when creating colorectal anastomosis given that the large intestine has a relatively vulnerable blood supply, making it more prone to developing segmental ischemia. Any disruption in these principles can lead to complications such as leak or stricture [2–4]. Intraoperative techniques such as white light endoscopy with visual subjective evaluation of perfusion and air leak tests are commonly employed to assess the integrity and completeness of bowel anastomoses.

More recently, the use of indocyanine green fluorescence angiography (ICG-FA) has been studied as a viable adjunct to help evaluate anastomotic tissue perfusion in real time. This has traditionally been used for serosal evaluation, but more recent studies have applied ICG-FA to mucosal assessment [5]. While evidence suggests that it may help reduce the rates of anastomotic leak in certain situations, follow-up studies have questioned its efficacy in this regard [6, 7]. Additionally, ICG-FA requires the injection of dye, which inherently carries a risk of allergic or adverse reactions, and ultimately the assessment of tissue perfusion relies on the operator’s qualitative judgment rather than objective data [8].

While ICG-FA provides real-time perfusion evaluation, oxygen saturation imaging (OSI) allows for quantitative, dye-free assessment of tissue oxygenation. OSI uses multispectral illumination to provide quantitative tissue oxygen saturation (StO_2_) measurements by differentiating oxy- and deoxyhemoglobin. Unlike ICG-FA, OSI requires no dye injection and provides measurable StO_2_ measurements that can be easily reproduced during the procedure. In recent years, OSI platforms have been applied to endoscopic imaging platforms. Another advantage of this technology in endoscopic platforms is that there is a minimal learning curve for proceduralists as it is compatible with standard endoscopic techniques. Initial feasibility studies evaluating oxygen saturation endoscopic imaging (OXEI) platforms have shown appropriate procedural safety and success [9–11]. Given that OXEI platforms are a relatively new development, there has been limited evaluation of their use in human subjects. As such, more data are needed to better assess the functionality of this technology and to better correlate StO_2_ levels with clinical outcomes. The aim of the current study is to evaluate mucosal tissue oxygen saturation using a novel OXEI platform in colorectal bowel anastomoses in human subjects. It is known that anastomostic staple lines, especially in colonic anastomoses, are ischemic in nature [12]. We hypothesized that the ELUXEO® VISION system will be able to detect a difference in StO_2_ at the anastomotic staple line compared to the baseline mucosa.

## Materials and methods

**Fig. 1:**
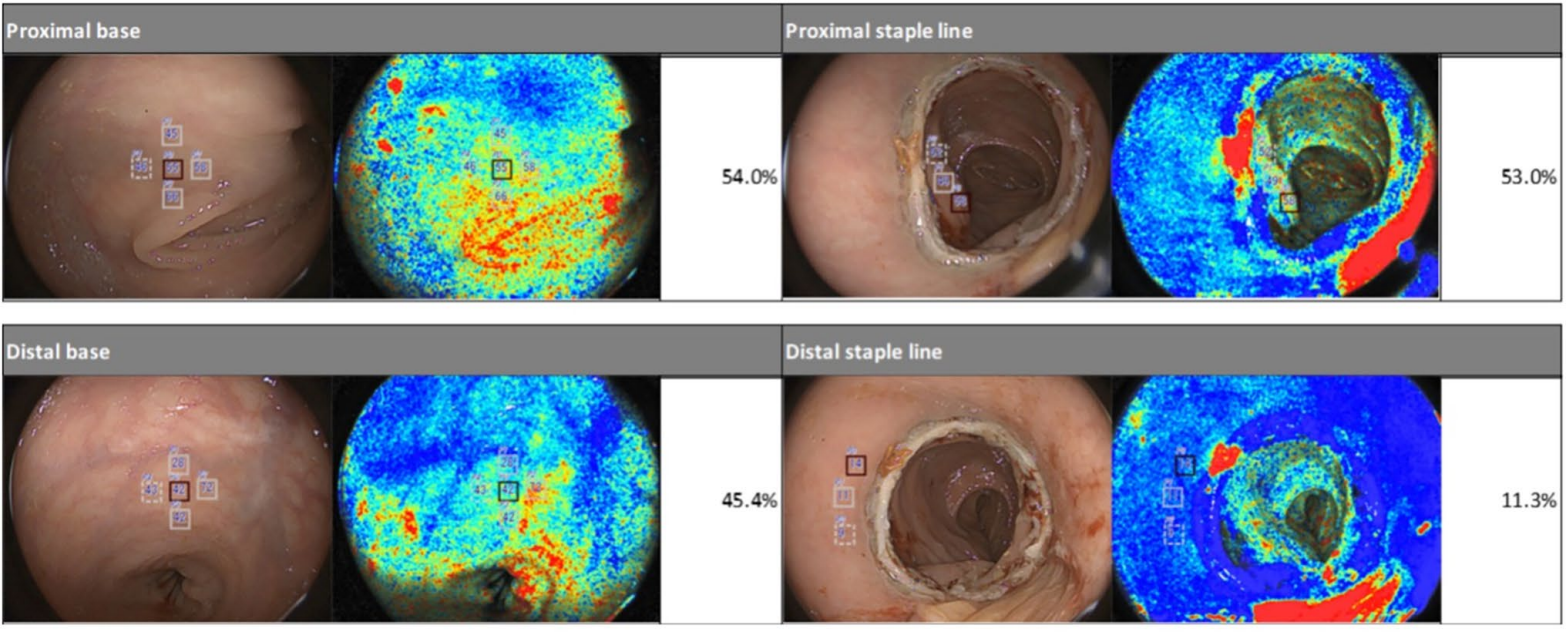
Endoscopic white light images (left) with superimposed StO_2_ map (right) using FUJIFILM ELUXEO® VISION System. The top photos represent the proximal staple line measurements, and the bottom photos represent proximal base measurements

We used the ELUXEO® VISION Endoscopic Imaging System with the EC-740 T/L colonoscope (FUJIFILM Corporation; Kanagawa, Japan) to obtain intraoperative, endoluminal bowel mucosal StO_2_ measurements after colon resection and anastomosis. This OXEI system uses multispectral illuminations and differing absorption properties of oxy- and deoxyhemoglobin to provide real-time numerical StO_2_ measurements. This OXEI platform superimposes a StO_2_ “map” onto a standard endoscopic image in real time and thereby augments traditional white light endoscopic evaluation with a more quantitative assessment of perfusion (Fig. 1).

This was a prospective, observational study of patients who underwent colorectal resection with primary anastomosis over a 3-month period. All patients enrolled were 18 years or older, were able to provide informed consent, and were undergoing planned laparoscopic colorectal resection and intestinal anastomosis. After anastomosis creation and at the time of routine intraoperative flexible sigmoidoscopy, mucosal StO_2_ measurements were taken endoscopically using the ELUXEO® VISION (EC-740 T/L colonoscope). Four measurements were taken: (1) proximal to the staple line (proximal base), (2) proximal edge of the staple line, (3) distal edge of the staple line, and (4) distal to the staple line (distal base). Calibrations were conducted at the proximal and distal bases. Endoscopic and laparoscopic procedures were recorded on videos, and stored still images were used to quantitatively evaluate the StO_2_ using analysis software developed by FUJIFILM Corporation at the completion of the study. Measurements were then recorded in a prospectively maintained database along with other operative findings and relevant clinical outcomes. Longitudinal data were obtained via chart review to track postoperative outcomes, and retrospective chart review was employed to gather other demographic data after patient enrollment.

Average and median StO_2_ measurements were calculated for each location 1 – 4 above. Differences between proximal and distal locations were calculated. Paired *t* tests were used to compare StO_2_ measurements between base and staple line, both proximal and distal to anastomosis. Given the small sample size, the analyses were based on the *t*-distribution.

## Results

A total of 12 patients were enrolled from June 2022 through September 2022. Among the 12 patients, the average age was 60.2 years (range 38 – 80), with seven males (58.3%). The average BMI was 26.8, and 11 patients were ASA class 2 or 3. The procedures included four laparoscopic sigmoid resections for diverticulitis (one with concurrent colovesical fistula), four low anterior resections for rectal cancer, three sigmoid colectomies for colon cancer, and one proctectomy and ileal-pouch anal anastomosis for ulcerative colitis. Details of the operative procedures chosen and associated StO_2_ measurements are shown in Table 1.

**Table 1:** StO2 measurements via endoscopic oxygen saturation imaging after intestinal anastomoses

**#**	Age	Gender	Diagnosis	Procedure	Prox. base (%)	Prox. staple line (%)	Diff. (%)	Distal base (%)	Distal staple line (%)	Diff. (%)
1	52	F	Diverticulitis	Lap sigmoidectomy	54.8	37.0	17.8	45.2	31.7	13.5
2	64	M	Diverticulitis	Lap sigmoidectomy	94.4	58.3	36.1	81.8	65.7	16.1
3	75	F	Diverticulitis	Lap sigmoidectomy	(33.4)	(0.0)^a^	(33.4)	32.4	19.7	12.7
4	75	F	Diverticulitis	Lap to open sigmoidectomy^b^	54.0	53.0	1.0	45.4	11.3	34.1
5	39	F	Colon CA	Lap sigmoidectomy	63.4	65.7	− 2.3	65.0	32.7	32.3
6	55	M	Colon CA	Open rectosigmoidectomy	54.8	67.0	− 12.2	83.4	81.3	2.1
7	64	M	Colon CA	Lap sigmoidectomy	61.4	54.3	7.1	46.2	34.7	11.5
8	38	M	Rectal CA	Lap LAR	46.8	20.7	26.1	(−)	(−)	(−)
9	66	M	Rectal CA	Lap LAR	59.0	51.3	7.7	40.4	37.3	3.1
10	70	M	Rectal CA	Lap LAR	(21.2)	(0.0) *	(21.2)	(−)	(−)	(−)
11	80	F	Rectal CA	Lap LAR	50.2	26.0	24.2	37.4	24.7	12.7
12	44	M	Ulcerative Colitis	Lap completion proctectomy w/ IPAA	43.0	22.7	20.3	(−)	(−)	(−)
Average					58.1	45.6	12.5	53.0	37.7	15.3
Sample standard deviation					14.2	17.6		19.0	22.2	
*T* value							2.681 (*p* = .025)			4.12 (*p* = .003)

^a^Excluded from analysis due to inconsistencies between StO_2_ readings and visual observations

^b^Intraoperative air leak

*F* female, *M* male, *CA* cancer, *Lap* laparoscopic, *LAR* low anterior resection *IPAA* ileal-pouch anal anastomosis, *Prox* proximal, *Diff* difference

The average and median mucosal StO_2_ measurements for all patients are as follows: average 53.0%, median 45.4%, range 32.4–83.4% at the distal base; average 37.7%, median 32.7%, range 11.3–81.3% at the distal staple line; average 45.6%, median 52.2%, range 20.7– 67.0% at the proximal staple line; and average 58.1%, median 54.8%, range 43.0– 94.4% at the proximal base. Due to inconsistencies between StO_2_ readings and visual observations, two patients were excluded from the proximal measurements and three from the distal measurements. The average StO_2_ difference was + 12.58% (95% CI 1.96 – 23.19%) between the proximal base and proximal staple line and + 15.34% (95% CI 6.76 – 23.9%) between the distal base and distal staple line. Statistically significant differences were found between well-perfused mucosa (base measurement) and the staple line, both proximal (*p *= 0.025) and distal (*p *= 0.003) to the anastomosis. Table 1 presents the individual StO_2_ results for each patient.

For all patients, white light endoscopy was performed in addition to OXEI, and there was no intraoperative evidence of anastomotic bleeding or anastomotic abnormalities observed via flexible sigmoidoscopy. Additionally, all patients had grossly “healthy appearing” mucosal edges at the site of anastomosis without overt evidence of ischemia as described in the operative report. Notably, one patient had a positive intraoperative air leak and an incomplete anastomotic donut. This patient had StO_2_ readings of 54.0% proximal base, 53.0% proximal staple line, and 1.0% proximal difference; 45.4% distal base, 11.3% distal staple line, and 34.1% distal difference. The distal measurements were lower than others in the cohort (base mean 54.1% [SD: 20.1], staple line mean 41.0% [SD: 21.3], and difference mean 13.0% [SD: 9.3]). This patient was successfully managed with a diverting loop ileostomy at the time of the index operation. The average length of surgery was 270.4 ± 110.5 min, with 10 of 12 patients’ surgeries taking longer than 180 min. The average estimated blood loss was 95 ml and only 1 patient required intraoperative blood transfusion. There were no intraoperative complications. Further details of the intraoperative data collected can be found in Table 2.

**Table 2:** Intraoperative characteristics and postoperative outcomes

Intraoperative characteristics	
Length of surgery, minutes, avg (SD)	270.4 (110.5)
Length of surgery > 180 min, *N* (%)	10 (83.3)
Estimated blood loss (%)	95 (88.9)
EBL > 200 ml, *N* (%)	3 (25)
Blood transfusion intra-OP, *N *(%)	1 (8.3)
Intra-OP complications, *N *(%)	0 (0)
Endoscopic findings, *N *(%)	
Anastomotic bleeding	0 (0)
Unhealthy mucosal edges at anastomosis	0 (0)
Anastomotic anomaly seen	0 (0)
Positive air leak	1 (8.3)
Incomplete donut	1 (8.3)
Revision of anastomosis	0 (0)

30-Day postoperative outcomes, *N* (%)	
Surgical site infection	1 (8.3)
Clinical anastomotic leak	0 (0)
Pulmonary embolus	0 (0)
ICU admission	1 (8.3)
Ileus	3 (25)
Complications	
Clavien–Dindo < III	6 (50)
Clavien–Dindo > III	0 (0)
Readmission	0 (0)
Reoperation	0 (0)

No patients developed clinically evident anastomotic leak within the first 30 days after surgery. One patient developed a surgical site infection, three developed ileus, and one was admitted temporarily to the intensive care unit postoperatively due to hypotension requiring short-term vasopressor support. Overall, six patients (50%) developed a minor postoperative complication within the first 30 days. All complications were Clavien–Dindo I or II, and no patients required reintervention/reoperation or had any 30-day readmission. Further details of the 30-day postoperative outcomes are also listed in Table 2.

## Discussion

In this study, endoluminal StO_2_ measurements taken with the ELUXEO® VISION system provided valuable insights into tissue perfusion at and near the anastomotic staple lines. The StO_2_ “maps,” which overlay oxygenation data onto standard white light images, revealed that the majority of StO_2_ values were lower near the anastomosis than in distant baseline mucosal areas. It is known that mucosal ischemia occurs after stapling of bowel [12, 13]. Therefore, this capability to detect subtle variations in perfusion, which are not easily visible through white light endoscopy alone, demonstrates the system’s ability to provide a more nuanced and objective view of tissue health.

Despite observing low StO_2_ values—down to 20.7% proximally and 11.3% distally—no patients in this study developed a clinical anastomotic leak, underscoring that even in low-perfusion areas, the tissue may still be viable in certain cases. Although the patient with the intraoperative air leak who was subsequently diverted had lower perfusion at the distal staple line site, we expect immediate air leaks to be from a technical error rather than a perfusion issue. This technology can complement traditional white light endoscopy and anastomotic leak tests by offering a quantitative assessment of anastomotic health. These added data allow surgeons to assess anastomotic perfusion, confirming its integrity or signaling the need for revision.

Because mucosal tissue is more sensitive to ischemia than serosal tissue, StO_2_ measurements offer a particularly informative view of mucosal oxygenation near the staple line—something not easily assessed with standard endoscopy. This enhanced visualization provides an early warning tool to reduce ischemia-related complications, enabling timely intervention based on real-time, quantitative perfusion data. Additionally, the VISION platform was shown to be safe, easy to use, and required minimal learning curve for colorectal surgeons. These findings suggest that VISION could be a valuable addition to intra- and perioperative care, offering an objective method for assessing anastomotic and intestinal perfusion.

In our study, there were inconsistencies between StO_2_ measurements and evaluation on traditional white light endoscopy in five patients. Several factors are known to influence oxygen saturation imaging, including motion artifact, angle of measurement, surface reflection or overlying substances, and peripheral oxygen saturation [14]. Additionally, device malfunction or user error could affect values obtained. Results should be interpreted appropriately and should augment traditional evaluation (i.e., visual inspection, white light endoscopy, air leak test) of an anastomosis rather than function as a replacement for these methods.

Further limitations of our study include inconsistency in calibration location and StO_2_ measurement site. The device was calibrated either proximal or distal to the anastomosis. Future studies will look to see if there is an association between the calibration site and StO_2_ values. When obtaining StO_2_ values, the “staple line” measurement was immediately adjacent to the staple line without incorporating the staples, and the “base” measurement was at least 5 cm from the staple line. Given this lack of standardization, reported StO_2_ values may have greater variability and represent a limitation to this study.

While OSI/OXEI systems have been shown to be feasible and safe for use in human subjects, as reflected in our current study, the clinical implications of StO_2_ measurements have not yet been elucidated. Despite recent advances in medical, surgical, and technological methods, anastomotic leak remains a serious complication following bowel anastomosis in colorectal surgery. Leak rates can range from 2 to 21%, with higher rates reported in more distal colorectal and coloanal anastomoses [2–4]. While the etiology of anastomotic leak can be multifactorial and is affected by patient comorbidities, prior surgical history, and perioperative treatments such as neoadjuvant chemotherapy and radiation, tissue perfusion remains an important factor [15]. In a recent study using the ELUXEO system to evaluate StO_2_ at esophageal anastomoses, it was shown that lower StO_2_ at the anastomosis was associated with a higher risk of leak [16]. There are limited data on using OXEI for the evaluation of colon anastomoses. Large-scale prospective studies with long-term follow-up are needed to establish the relationship between StO_2_ measurements and clinical outcomes, such as anastomotic leak or stricture, to determine clinically relevant StO_2_ cutoffs. Given our study had no anastomotic leaks, more studies may help answer this question. Additional studies are currently underway at our institution to evaluate these outcomes and the application of OSI technology in both endoscopy and laparoscopy. Our future study aims to determine the best outcome measure for a healthy anastomosis (i.e., whether anastomotic complications are associated with an absolute minimum StO_2_ measurement, a percentage difference in StO_2_ relative to nearby perfused mucosa, etc.). For future studies, we hypothesize that higher perfusion and increased StO_2_ scores in bowel anastomosis may lead to decreased leak rates.

## Supplementary Information

Below is the link to the electronic supplementary material.

Supplementary file1 (DOCX 33 kb)

Supplementary file1 (DOCX 33 kb)

## References

[1] Nandakumar G, Stein SL, Michelassi F (2009) Anastomoses of the lower gastrointestinal tract. Nat Rev Gastroenterol Hepatol 6(12):709–716. 10.1038/nrgastro.2009.185 19884894 10.1038/nrgastro.2009.185

[2] Paun BC, Cassie S, MacLean AR, Dixon E, Buie WD (2010) Postoperative complications following surgery for rectal cancer. Ann Surg 251(5):807–818. 10.1097/SLA.0b013e3181dae4ed 20395841 10.1097/SLA.0b013e3181dae4ed

[3] Midura EF, Hanseman D, Davis BR et al (2015) Risk factors and consequences of anastomotic leak after colectomy: a national analysis. Dis Colon Rectum 58(3):333–338. 10.1097/dcr.0000000000000249 25664712 10.1097/DCR.0000000000000249

[4] McDermott FD, Heeney A, Kelly ME, Steele RJ, Carlson GL, Winter DC (2015) Systematic review of preoperative, intraoperative and postoperative risk factors for colorectal anastomotic leaks. Br J Surg 102(5):462–479. 10.1002/bjs.9697 25703524 10.1002/bjs.9697

[5] Amagai H, Miyauchi H, Muto Y et al (2020) Clinical utility of transanal indocyanine green near-infrared fluorescence imaging for evaluation of colorectal anastomotic perfusion. Surg Endosc 34(12):5283–5293. 10.1007/s00464-019-07315-7 31820154 10.1007/s00464-019-07315-7

[6] Shen R, Zhang Y, Wang T (2018) Indocyanine green fluorescence angiography and the incidence of anastomotic leak after colorectal resection for colorectal cancer: a meta-analysis. Dis Colon Rectum 61(10):1228–1234. 10.1097/dcr.0000000000001123 30192332 10.1097/DCR.0000000000001123

[7] Jafari MD, Pigazzi A, McLemore EC et al (2021) Perfusion assessment in left-sided/low anterior resection (PILLAR III): a randomized, controlled, parallel, multicenter study assessing perfusion outcomes with PINPOINT near-infrared fluorescence imaging in low anterior resection. Dis Colon Rectum 64(8):995–1002. 10.1097/dcr.0000000000002007 33872284 10.1097/DCR.0000000000002007

[8] Jiao Y, Liu Y, Jin M (2024) Exploring the dark side of diagnostic dyes with a focus on indocyanine green’s adverse reactions. Sci Rep 14(1):30155. 10.1038/s41598-024-81903-z 39627439 10.1038/s41598-024-81903-z PMC11614906

[9] Kaneko K, Yamaguchi H, Saito T et al (2014) Hypoxia imaging endoscopy equipped with laser light source from preclinical live animal study to first-in-human subject research. PLoS ONE 9(6):e99055. 10.1371/journal.pone.0099055 24915532 10.1371/journal.pone.0099055 PMC4051687

[10] Saito T, Yamaguchi H (2015) Optical imaging of hemoglobin oxygen saturation using a small number of spectral images for endoscopic application. J Biomed Opt 20(12):126011. 10.1117/1.jbo.20.12.126011 26720878 10.1117/1.JBO.20.12.126011

[11] Hasegawa H, Takeshita N, Ito M (2020) Novel oxygen saturation imaging endoscopy to assess anastomotic integrity in a porcine ischemia model. BMC Surg 20(1):250. 10.1186/s12893-020-00913-6 33092548 10.1186/s12893-020-00913-6 PMC7583199

[12] Hanberg P, Bue M, Thomassen M et al (2021) Influence of anastomoses on intestine ischemia and cefuroxime concentrations: evaluated in the ileum and colon in a porcine model. World J Gastrointest Pathophysiol 12(1):1–13. 10.4291/wjgp.v12.i1.1 33585069 10.4291/wjgp.v12.i1.1 PMC7852486

[13] Myers C, Mutafyan G, Petersen R, Pryor A, Reynolds J, Demaria E (2009) Real-time probe measurement of tissue oxygenation during gastrointestinal stapling: mucosal ischemia occurs and is not influenced by staple height. Surg Endosc 23(10):2345–2350. 10.1007/s00464-009-0342-5 19263155 10.1007/s00464-009-0342-5

[14] Alomari M, Wadiwala I, Bowers S, Elli EF, Thomas M (2024) Oxygen saturation endoscopic imaging as a novel alternative to assess tissue perfusion during esophagectomy. Surg Innov 31(6):622–626. 10.1177/15533506241290071 39361295 10.1177/15533506241290071

[15] Matthiessen P, Hallböök O, Andersson M, Rutegård J, Sjödahl R (2004) Risk factors for anastomotic leakage after anterior resection of the rectum. Colorectal Dis 6(6):462–469. 10.1111/j.1463-1318.2004.00657.x 15521937 10.1111/j.1463-1318.2004.00657.x

[16] Fujita T, Sato K, Ozaki A et al (2022) A novel imaging technology to assess oxygen saturation of the gastric conduit in thoracic esophagectomy. Surg Endosc 36:7597–7606 35364701 10.1007/s00464-022-09199-6

