# Supplementary material for: Real-time bowel perfusion monitoring with FUJIFILM ELUXEO® VISION endoscopic imaging system in colorectal surgery
Source: Surg Endosc. 2025 Dec 29;40(2):1704–10. doi: 10.1007/s00464-025-12526-2 (PMC12880988; doi:10.1007/s00464-025-12526-2)
Supplement: Supplementary file 1 — Supplementary file1 (DOCX 33 kb) [file 464_2025_12526_MOESM1_ESM.docx]

**Dynamic Video Narration**

Anastomotic leak is a serious complication ranging from 2-21% and is associated with both patient discomfort, longer hospital stays, and increased hospital cost. While the etiology of anastomotic leak can be multifactorial, sound surgical technique reduces the risk. ICG is the most widely used way to assess tissue perfusion; however, it is associated with allergic reactions and doesn't give us a quantitative judgement.

The Fujifilm ELUXEO platform allows surgeons to obtain real-time, intraoperative bowel mucosal StO₂ measurements. The aim of our study was to evaluate mucosal tissue oxygen saturations in colorectal anastomoses using the platform. We hypothesized that the platform would detect a meaningful difference in StO₂ measurements between the staple line and proximal bowel. A secondary objective was to assess the safety and feasibility of using the Fujifilm ELUXEO platform during colorectal surgery.

We conducted a three-month prospective observational study measuring StO_2_ measurements at the proximal base, proximal staple line, distal staple line, distal base. The EMR was reviewed to track postoperative outcomes.

We enrolled a total of 12 patients between June and September of 2022.  The average age was just over 60 years, and most patients were ASA class 2 or 3. Procedures included sigmoid resections for diverticulitis, low anterior resections for rectal cancer, sigmoid colectomies for colon cancer, and one proctectomy with ileal-pouch anal anastomosis. Using the Fujifilm ELUXEO platform, we measured mucosal StO₂ at four locations: the proximal and distal bases, and the proximal and distal staple lines. Across all patients, we consistently saw higher oxygen saturations at the bases compared to the staple lines. On average, the proximal base had about a 12.5% higher StO₂ than the proximal staple line, and the distal base had roughly a 15% higher StO₂ than the distal staple line. Both differences were statistically significant. A few measurements were excluded due to inconsistencies with visual assessment, and full individual results are shown in this table.

For 30-day postoperative outcomes, we observed low complication rates across the cohort.  There was one surgical site infection, representing about eight percent of patients, and no clinical anastomotic leaks.  We had no PEs, and there was one ICU admission, again about eight percent. Three patients, or twenty-five percent, developed postoperative ileus. Overall complications were mild: six patients had Clavien–Dindo class II or lower, and there were no Clavien–Dindo grade III or higher complications.  There were also no readmissions and no reoperations within thirty days. More detailed data, including full ranges, medians, and confidence intervals are available in the manuscript

The Fujifilm ELUXEO platform demonstrates both safety and feasibility in this study. We observed statistically significant differences in StO₂ values between the bowel bases and the staple line. However, larger-scale prospective studies are needed to determine how these StO₂ measurements relate to clinical outcomes.

Here we’re using the Fujifilm oxygen saturation endoscopic imaging, or OXEI, to assess real-time bowel perfusion. You’ll notice the heatmap in the lower right corner with red indicating adequate blood flow and blue indicating low oxygen saturation. At the anastomosis site, blood flow is absent just as expected in this region. Continuing both proximally and distally to the anastomosis, the heatmap once again shows robust blood flow. This imaging technique helps ensure precise assessment of tissue perfusion throughout the procedure. Intraoperative techniques such as white light endoscopy and air leak tests play a critical role in assessing the integrity and completeness of bowel anastomoses. To maximize accuracy, the assessment of tissue perfusion should rely on objective data rather than subjective judgement, providing a clear, measurable foundation to guide clinical decisions and enhance patient care. This objective data can potentially be provided with advanced devices like the ELUXEO. These techniques are invaluable for identifying and ensuring proper tissue perfusion – a fundamental component of successful endoluminal and laparoscopic procedures.

This device can overlay a heatmap to highlight areas of low oxygen saturation, as shown here at the anastomosis site. Multiple images can be captured throughout the procedure for continuous monitoring. A key advantage of this platform is its compatibility with standard endoscopic techniques, meaning there is minimal learning curve for proceduralists. Initial safety and feasibility studies on the oxygen saturation endoscopic imaging (OXEI) platform have demonstrated both procedural safety and effectiveness. The endoscope also allows us to easily toggle between modes to meet our assessment needs during surgery. As we look to the future, real-time, dye-free, quantitative perfusion assessment at the anastomosis holds the potential to significantly impact patient outcomes. A study to evaluate this approach is currently underway at our institution, paving the way for advancements in patient care and surgical precision.

Thank you for joining us as we continue to explore innovations that optimize outcomes and elevate standards in surgical practice.
